# Chronic non-discriminatory social defeat stress reduces effort-related motivated behaviors in male and female mice

**DOI:** 10.1038/s41398-021-01250-9

**Published:** 2021-02-15

**Authors:** Andrew Dieterich, Tonia Liu, Benjamin Adam Samuels

**Affiliations:** 1 Neuroscience Graduate Program, Rutgers University, Piscataway, 08854 NJ USA; 2 Department of Psychology, Behavioral and Systems Neuroscience, Rutgers University, Piscataway, NJ 08854 USA

**Keywords:** Neuroscience, Psychology

## Abstract

Reward and motivation deficits are prominent symptoms in many mood disorders, including depression. Similar reward and effort-related choice behavioral tasks can be used to study aspects of motivation in both rodents and humans. Chronic stress can precipitate mood disorders in humans and maladaptive reward and motivation behaviors in male rodents. However, while depression is more prevalent in women, there is relatively little known about whether chronic stress elicits maladaptive behaviors in female rodents in effort-related motivated tasks and whether there are any behavioral sex differences. Chronic nondiscriminatory social defeat stress (CNSDS) is a variation of chronic social defeat stress that is effective in both male and female mice. We hypothesized that CNSDS would reduce effort-related motivated and reward behaviors, including reducing sensitivity to a devalued outcome, reducing breakpoint in progressive ratio, and shifting effort-related choice behavior. Separate cohorts of adult male and female C57BL/6 J mice were divided into Control or CNSDS groups, exposed to the 10-day CNSDS paradigm, and then trained and tested in instrumental reward or effort-related behaviors. CNSDS reduced motivation to lever press in progressive ratio and shifted effort-related choice behavior from a high reward to a more easily attainable low reward in both sexes. CNSDS caused more nuanced impairments in outcome devaluation. Taken together, CNSDS induces maladaptive shifts in effort-related choice and reduces motivated lever pressing in both sexes.

## Introduction

Major depressive disorder (MDD) is a prevalent and costly psychiatric disorder that affects more than 10% of the population and can be precipitated by chronic exposure to stressors^1–4^. Approximately half of those diagnosed with MDD suffer from anhedonia, and reward processing deficits occur in many other disorders including schizophrenia and substance abuse disorder^5,6^. Importantly, deficits in reward processing can be effectively studied using analogous behavioral tests in both humans and rodents^7^. Thus, studying individual behavioral components of reward function in rodents exposed to chronic stress should help improve our understanding of the etiology of psychiatric disorders. However, preclinical mood disorder research has historically focused on avoidance behaviors that were interpreted as measuring anxiety-like behavior or on antidepressant-sensitive behaviors such as forced swim test in rodents^8^.

Reward learning and responsiveness to social or monetary rewards is impaired in MDD patients^9–11^. fMRI of MDD patients performing reward tasks suggests these maladaptive impairments may be due in part to reduced activation in neural circuitry such as the nucleus accumbens^12,13^. In progressive ratio tasks where increasing amounts of effort are required for subsequent rewards, MDD patients exhibit significantly less motivation and rewards earned^14^. Likewise, in an effort-expenditure for rewards task (EEfRT) where participants have the option between low effort/low monetary reward and high effort/high monetary reward tasks, MDD patients are less likely to choose the high effort option^15^. The likelihood of choosing higher effort/higher reward options in EEfRT negatively correlates with self-reported anhedonia^16^.

Chronic life stressors can precipitate mood disorders such as MDD, but the effects of stress on reward processing and motivation may be different between men and women^17^. MDD is diagnosed more frequently in women than in men^3,18^, and preclinical rodent work has historically excluded females^19^. For example, two commonly used rodent chronic stress paradigms, chronic corticosterone (CORT) administration and chronic social defeat stress (SDS), were developed in male mice and are less effective in female mice. The chronic CORT model effectively increases maladaptive avoidance behaviors in male but not in female C57BL/6 J mice^20–23^. Thus, it is unknown whether there are any sex differences in how chronic stress alters reward and motivation behaviors.

Recent SDS variants have permitted the study of how chronic stress alters behavior in female mice but require either complex stereotaxic surgeries or urine collection from additional CD-1 mice and then application of this urine onto the females in each daily session of defeat^24,25^. Female to female aggression is also present in the California mouse, but not in more widely used laboratory strains like C57BL/6 J^26^. However, one recent variant of social defeat, chronic non-discriminatory social defeat stress (CNSDS), which simultaneously exposes a male and a female C57BL/6 J to an aggressive conspecific, produces robust effects in avoidance and other affective behavioral tasks, including a relatively minor effect in sucrose preference, a common behavioral measure of anhedonia^27^. Therefore, CNSDS may provide a way to determine if there are sex differences in how chronic stress affects reward and motivation.

In this study we examined how CNSDS affects instrumental reward behaviors and effort-related choice behavior in both male and female mice. We hypothesized that CNSDS would reduce sensitivity to a devalued outcome, reduce motivation to lever press in progressive ratio, and shift effortful responding from a high effort/high reward outcome to a low effort/low reward outcome in both sexes.

## Materials and methods

### Animals

60 adult male and 60 adult female C57BL/6 J mice (Jackson Laboratories, Bar Harbor, ME), and 60 retired male breeder CD-1s (Charles River Laboratory, Wilmington, MA) were used in these experiments. All mice were housed in a dedicated animal colony room on a 12 L:12D light/dark (6:00 AM lights on: 6:00 PM lights off) schedule and maintained at ~60% humidity and 72°F. All experiments were approved by the Institutional Animal Care and Use Committee (IACUC) at Rutgers University and conducted in compliance with NIH animal care guidelines. Behavior testing was conducted between 8:00 AM and 3:00 PM daily, with the regular room lights on.

### Chronic Non-Discriminatory Social Defeat Stress (CNSDS)

Adult male and female C57BL/6 J mice (Jackson Laboratories, Bar Harbor, ME) were randomly assigned to control or CNSDS conditions, and then completed Control or CNSDS protocols as previously described^27,28^. In the control condition, a male and female C57BL/6 J mouse interacted for 5 min daily, followed by co-housing with a perforated Plexiglas cage divider. In the CNSDS condition, a male and female C57BL/6 J mouse were placed in the home cage of a retired breeder CD-1 male (Charles River Laboratory, Wilmington, MA) for 10 consecutive daily 5-minute defeat sessions. After each defeat session, the male C57BL/6 J mouse was co-housed with a novel CD-1 male, separated by a cage divider. The female was co-housed with the aggressor CD-1, again separated by a cage divider but allowing sensory interaction, but no physical contact. Co-housing alternated each day. Thus, the male and female pair were each housed with the attacking CD-1 on half of CNSDS days. Following CNSDS, mice were pair-housed in standard clear Plexiglas mouse cages with corncob bedding with perforated Plexiglas separating the pair of mice in the cage. Following CNSDS, all mice were food-deprived and maintained at 90% of their free-feeding body weight and fed daily with standard lab chow at least 1-hour after behavior testing.

### Instrumental conditioning

In the instrumental reward behavior cohort, all mice were trained to lever press and tested in reward-related behaviors in standard mouse instrumental conditioning chambers (Med Associates, Fairfax, VT), connected via a power control and interface unit to a dedicated computer with MED-PC IV software (Med Associates, Fairfax, VT) running custom scripts. Each instrumental chamber consisted of a single retractable response lever with a reward port delivering 20 mg dustless precision food pellet reinforcers (Bio-Serv, Flemington, NJ), connected by Y-tubing to pellet hoppers.

Control males (*n* = 20) and females (*n* = 20), and CNSDS-exposed males (*n* = 20) and females (*n* = 20) were first exposed to instrumental responding on a Fixed Ratio 1 (FR1) schedule of reinforcement, where each lever press is reinforced. A single active lever was ejected into the chamber at the start of each trial, and was retracted following a lever press, coinciding with a single reward pellet being delivered into the reward port for consumption. Following FR1 sessions, mice completed a Variable Ratio 2 (VR2) session, where every 1, 2, or 3 lever presses was reinforced. At this point, all mice had lever pressed more than at least 20 times in a FR1 or VR2 session, and mice progressed to outcome devaluation followed by progressive ratio testing. No mice were excluded from these analyses.

### Outcome devaluation

Mice then completed a satiety-based outcome devaluation procedure. In one session (“devalued”), mice were pre-fed with reinforcer pellets in the home cage 1-hour prior to a 5-minute extinction test where the lever was ejected and responses recorded, but no reinforcers were delivered. A day later, mice completed a single VR2 session to re-acquire the response–reward relationship. In the other outcome devaluation session (“valued”), mice were pre-fed with standard lab chow in the home cage 1-hour prior to an identical 5-minute extinction test. Response in both “devalued’ and “valued” sessions were measured and compared to determine the effect of CNSDS on responding in both sessions.

### Progressive ratio

Mice were tested in a 1-hour progressive ratio (PROG) session, where increasingly greater numbers of lever presses were required for reinforcement. The PROG ratio schedule increased on a linear X + 3 scale (3, 6, 9, X + 3). Lever presses, reinforcers earned, and final ratio reached were recorded in the 1-hour PROG test session.

### Y-maze barrier task

A Y-maze barrier mouse version^28,29^ of the rat T-Maze barrier task^30–33^ was implemented to assess the effect of CNSDS in male and female C57BL/6 J mice on an effort-related choice behavior^29^. Control (*n* = 10) and CNSDS (*n* = 10) males, and Control (*n* = 9) and CNSDS (*n* = 10) female C57BL/6 J mice completed respective control or CNSDS paradigms, followed by being food-deprived, habituated to and trained in the Y-maze task, and then tested at successively taller barrier heights. One female in the Control group died during training and was excluded from analyses. The Y-maze consisted of a white Plexiglas maze of a start box and two reward arms, with 20 cm high walls, 26 cm long arms, and a uniform width of 7 cm. When a Plexiglas barrier was removed in the start box, the mouse could traverse the Y-maze and enter either arm. Either 2 pellets or 4 pellets (20 mg dustless precision reinforcer pellets, Bio-Serv, Flemington, NJ), were placed in small food dishes at the end of the low reward (LR) and high reward (HR) arms, respectively, and were counterbalanced between mice in each group.

### Y-maze barrier task testing

Mice were first trained to discriminate between high (HR) and low (LR) reward arms in daily session of 5 trials, followed by two days of forced-choice sessions, consisting of 10 alternating forced-choice trials where one arm was blocked off. Mice then were tested in free-choice sessions, which began with two forced-choice trials, followed by 10 free-choice trials. In each free choice trial, mice had one minute to consume the pellets, before it was removed to the home cage. The mice were cycled in the Y-maze so that there was on average a 15-minute inter-trial interval. Mice were trained in these free-choice sessions until they reached the criterion of >70% HR arm selection. Following free choice training in the Y-maze, mice completed 3 daily sessions with 10 cm, 15 cm, or 20 cm barriers placed into the HR arm. Upon completing Y-maze barrier testing, all mice completed a control condition where 10 cm barriers were placed in both HR and LR arms, to examine if reward discrimination or barrier climbing ability is affected by CNSDS or differs by sex. All HR or LR arm selections and latencies to enter the arm were recorded for each trial in all sessions.

### Free feeding test

Following 18 h of food deprivation, a single food pellet of standard lab chow was placed in the home cage of each mouse for both cohorts following all instrumental or Y-maze behavior testing, for a 60-minute free-feeding behavior and the weight of food consumed (g) was recorded.

### Open field test

To assess avoidance behavior as well as overall locomotion, a 10-minute open field test was conducted for all mice in both cohorts following behavioral testing. Mice were placed into the corner of the open field chamber (43 × 43 cm) and allowed to explore for 10 min. Motor Monitor (Kinder Scientific, Poway, CA) detected beam breaks with infrared photobeams surrounding the open field. Time spent in the center of the open field (11 × 11 cm), as well as overall distance traveled were recorded.

### Estrous cycle

Vaginal lavage was conducted as previously described^34,35^, in order to determine which stage of the estrous cycle each female was in during test sessions (Supplemental Fig. 1). Following all behavioral testing for the day, 100 *u*l of ddH_2_O was pipetted onto the surface of the vaginal opening for each female and then pipetted onto microscope slides (Superfrost Microscope Slides, Fisher Scientific, Waltham, MA) which were imaged using brightfield microscopy (Evos FL Auto 2 Imaging System, Thermo Fisher, Waltham, MA) at 10X magnification. Estrous cycle stages were determined based on the presence of large nucleated cells as proestrus, large cornified cells as estrus, the presence of leukocytes and also rounder un-nucleated cells characterized metestrus, and larger leukocytes with some cornified cells or epithelial cells also present characterized diestrus^36^.

### Data analysis and statistics

Parametric hypotheses about the effects of CNSDS on reward and effort-related choice behaviors were assessed using parametric analyses, including 2 × 2 ANOVAs with Tukey post hoc tests with CNSDS and sex as between-subjects factors, or unpaired *t*-tests where appropriate, including when examining males and females separately. In one cohort, Control (*n* = 20) and CNSDS (*n* = 20) males and Control (*n* = 20) and CNSDS (*n* = 20) females were tested in instrumental reward behaviors. In a separate cohort, Control (*n* = 10) and CNSDS (*n* = 10) male and Control (*n* = 9) and CNSDS (*n* = 10) female mice were tested in the Y-maze barrier task. For latency to select HR and LR arms in the Y-maze barrier task, while latency was recorded for all trials in all Free Choice, 10 cm, 15 cm, 20 cm, and Discrimination sessions, many mice almost entirely selected the HR arm in Free Choice sessions (Supplemental Fig. 2A), or LR arm in 15 cm (Supplemental Fig. 2C), and 20 cm (Supplemental Fig. 2D) barrier testing sessions. Therefore, only 10 cm barrier session (Supplemental Fig. 2B) and Discrimination session (Supplemental Fig. 2E) latencies were parametrically examined (2 × 2 x 2 ANOVA). All analysis and statistics were performed in GraphPad Prism 7 (GraphPad Software, San Diego, CA). Significance was determined at *p* < 0.05.

## Results

### Effects of CNSDS on instrumental behaviors

**Fig. 1:**
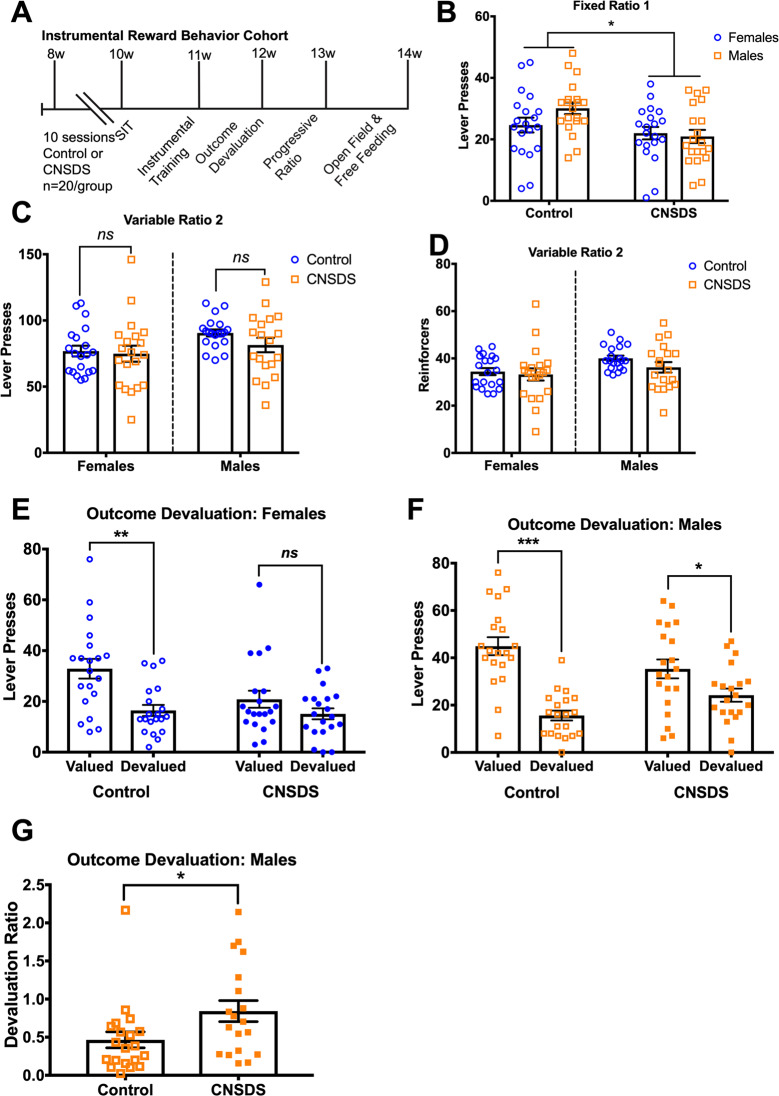
CNSDS impairs instrumental responding and outcome devaluation. **A** Experimental timeline of the CNSDS protocol followed by fixed ratio 1 (FR1), outcome devaluation, progressive ratio, and free-feeding and open field behavior tests in Control and CNSDS male and female mice (*n* = 20/group). **B** In a Fixed Ratio 1 (FR1) session, CNSDS mice lever pressed less than Control mice. **C** In a Variable Ratio 2 (VR2) session, collapsed by CNSDS, males lever pressed more than females, but separated by sex there are no differences between groups. **D** Reinforcers earned in the VR2 session are similar between groups. **E** In Outcome Devaluation, Control but not CNSDS females reduced Devalued lever presses compared to that in the Valued session. **F** Control males substantially reduced lever presses in the Devalued session compared to Valued session, and CNSDS males similarly reduced presses, though to a lesser degree. **G** However, post hoc analysis of devaluation ratio (devalued presses divided by valued presses) indicate CNSDS males have a significantly higher ratio than Control, similarly indicating that CNSDS reduces sensitivity for an outcome that has been devalued. Bars are mean ± SEM. *p < 0.05; **p < 0.01; ***p < 0.001.

We began by assessing how CNSDS affects instrumental reward behaviors in both sexes (Fig. 1A). C57BL6/J mice were divided into Control or CNSDS by sex (*n* = 20/group). In CNSDS, male and female C57BL6/J mice are simultaneously exposed to a pre-screened aggressive CD-1 mouse for 5 min per day over 10 days^27,28^. CNSDS did not affect body weight in males or in females (Supplemental Fig. 3A). CD-1 aggressions were averaged for each mouse, and similar to our original CNSDS report^27^, an independent samples *t*-test indicated that CNSDS males were aggressed more frequently than females (*t*(38) = 13.26, *p* < 0.0001) (Supplemental Fig. 3B).

Next, all mice were trained to lever press in standard mouse instrumental conditioning chambers. Lever presses across 7 daily FR1 sessions showed that all groups of mice gradually increased lever pressing across sessions (Supplemental Fig. 3C). We analyzed lever presses in the final Fixed Ratio 1 (FR1) session by two-way ANOVA with stress (Control; CNSDS) and sex (male; female) as between-subjects factors. We observed a main effect of CNSDS (*F*(1, 76) = 7.7, *p* = 0.0069), but no main effect of sex (*F*(1, 76) = 0.1071, *p* = 0.3041) or interaction (*F*(1, 76) = 2.337, *p* = 0.1305) (Fig. 1B). Body weights did not correlate with lever presses in the final FR1 session (Supplemental Figure 4A). Thus, CNSDS impairs lever pressing on a FR1 schedule of reinforcement.

Mice then completed a Variable Ratio 2 (VR2) session where every 1, 2, or 3 lever presses was reinforced^21^. A two-way ANOVA with stress (Control; CNSDS) and sex (male; female) as between-subjects factors revealed a main effect of sex in the number of lever presses (*F*(1, 76) = 4.652, *p* = 0.03423) and in the number of reinforcers earned (*F*(1, 76) = 5.015, *p* = 0.0281). Importantly, we also saw a significant correlation between body weight and reinforcers earned in these VR2 sessions (*r* = 0.2313, *p* = 0.0403; Supplemental Fig. 4B). Thus, we analyzed males and females separately for these VR2 sessions. Separate unpaired *t*-tests within each sex showed no effect of CNSDS on lever presses in either females (*t*(38) = 0.275, *p* = 0.7848) or in males (*t*(38) = 1.545, *p* = 0.1309) (Fig. 1C). Likewise, for number of reinforcers earned in VR2, separate unpaired *t*-tests showed no effect of CNSDS in either females (*t*(38) = 0.4036, *p* = 0.6888) or in males (*t*(38) = 1.563, *p* = 0.1266) (Fig. 1D). Thus, when examined separately, CNSDS does not reduce lever presses or reinforcers earned in VR2 in males or females.

We next assessed how CNSDS affects reward valuation in both males and females by testing mice in a satiety-based outcome devaluation procedure. We examined lever presses in both valued and devalued sessions. A three-way repeated measures mixed ANOVA between CNSDS (Control; CNSDS), sex (Male; Female), and outcome devaluation session (valued; devalued) revealed main effects of sex (*F*(1, 76) = 15.39, *p* < 0.0001) and a significant session x sex interaction (*F*(1, 76) = 4.229, *p* = 0.0414). Importantly, body weights were significantly correlated with lever presses in the devalued session (*r* = 0.2276, *p* = 0.0424) and showed a trend to correlate in the devalued session (*r* = 0.2150, *p* = 0.0555; Supplemental Fig. 4C, D). Therefore, we analyzed males and females separately in outcome devaluation by two-way ANOVA. Within females, there was a significant main effect of devaluation (*F*(1, 38) = 18.35, *p* = 0.0001) and a significant stress x session interaction (*F*(1, 38) = 4.263, *p* = 0.0458) (Fig. 1E). Control females (*p* = 0.0001), but not CNSDS females (*p* = 0.2342), reduced lever presses in the devalued compared to the valued session. For males, there was a significant main effect of devaluation (*F*(1, 38) = 36.62, *p* < 0.0001) and a significant stress x session interaction (*F*(1, 38) = 7.425, *p* = 0.0097) (Fig. 1F). Control males (*p* < 0.0001) and CNSDS males (*p* = 0.0473) both reduced lever presses in the devalued session compared to the valued session, although this measure appeared attenuated in CNSDS males. Given the significant stress x session interaction, we next examined the ratio of devalued to valued presses in males post hoc. An unpaired *t*-test (*t*(38) = 2.188, *p* = 0.0351) confirmed that CNSDS attenuated sensitivity for a devalued outcome in male mice (Fig. 1G). These observations indicate nuanced effects where CNSDS abolishes sensitivity to a devalued outcome in females but attenuates sensitivity in males.

**Fig. 2:**
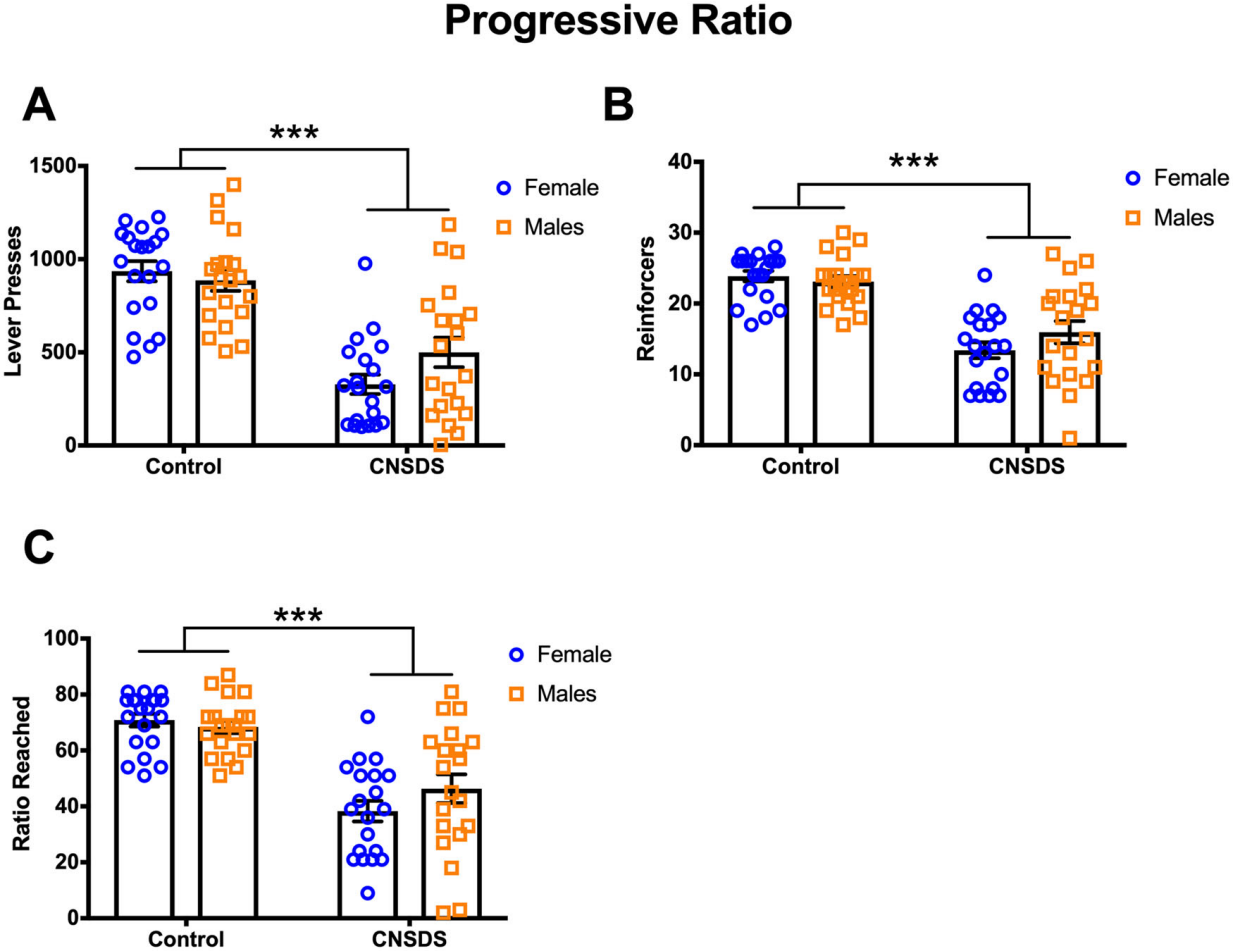
CNSDS reduces motivation in progressive ratio. **A** Lever presses in Control and CNSDS males and females in a 1-hour progressive ratio (PROG) session, where the number of presses required for each subsequent reinforcer increased linearly (3, 6, 9, 12, X + 3). Collapsed by sex, CNSDS reduced lever presses in PROG. **B** Reinforcers earned in the PROG session is less in CNSDS mice than in Controls. **C** Final PROG ratio reached is similarly reduced in CNSDS mice compared to Control. Bars are mean ± SEM. *p < 0.05; **p < 0.01; ***p < 0.001.

We next wanted to assess whether CNSDS affects motivation. To this end we exposed mice to a progressive ratio (PROG) test, where the number of lever presses required for each successive reinforcer increased until a mouse stopped lever pressing for 5 min or 1 h had passed (Fig. 2). Total number of lever presses, reinforcers earned, and ratio reached were recorded in the PROG test session. For lever presses made during the PROG test session, a two-way ANOVA with sex and stress as between-subjects factors revealed a main effect of stress (*F*(1, 76) = 65.5, *p* < 0.0001), but no main effect of sex (*F*(1, 76) = 0.99, *p* = 0.3229) or interaction (*F*(1, 76) = 3.229, *p* = 0.0763) (Fig. 2A). For PROG reinforcers earned, there was a main effect of stress (*F*(1, 76) = 63.35, *p* < 0.0001), but no main effect of sex (*F*(1, 76) = 0.6626, *p* = 0.4182) or interaction (*F*(1, 76) = 2.227, *p* = 0.1397) (Fig. 2B). For PROG ratio reached, there was a main effect of stress (*F*(1, 76) = 59.75, *p* < 0.0001), but no main effect of sex (*F*(1, 76) = 0.6387, *p* = 0.4267) or interaction (*F*(1, 76) = 2.185, *p* = 0.1435) (Fig. 2C). Body weight did not correlate with PROG reinforcers earned (Supplemental Fig. 4E). Taken together, CNSDS robustly reduced lever presses, reinforcers earned, and final ratio reached in a 1-hour linearly increasing (X + 3) PROG test. Thus, in both males and females, CNSDS impairs motivation to expend effort for a reinforcer as the number of lever presses required to obtain each successive reinforcer is steadily increased.

To confirm that the results in outcome devaluation and progressive ratio were due to CNSDS-induced deficits in reward valuation and motivation, we next assessed free feeding behavior. In a 1-h free feeding task, Control and CNSDS males and females were food-deprived for 18 h and then placed in a fresh cage with one food pellet of standard lab chow for consumption. Consumption was then assessed and adjusted by body weight. Females consumed more food per gram body weight compared to males, but CNSDS had no effect on free feeding (Supplemental Fig. 3D). Lastly, we ran a 10-minute Open Field test (OFT) to assess exploratory activity. Similar to our previous report^27^, CNSDS increased avoidance of the aversive center of the open field in both sexes but did not affect overall distance traveled (Supplemental Fig. 3E, F).

We also tracked estrous cycle in the females in Fixed Ratio 1, outcome devaluation, progressive ratio, and free feeding (Supplemental Fig. 1). Similar to our previous study on avoidance behaviors^27^, we saw no effects of estrous stage on any measures assessed in the reward and motivation behaviors. There were some nuanced differences in the free feeding task, where CNSDS females in the diestrus phase of the estrous cycle consumed less compared to CNSDS females in proestrus (*p* = 0.0432) or CNSDS females in metestrus (*p* = 0.0031) (Supplemental Fig. 1). We also assessed FR1, outcome devaluation, progressive ratio, and free feeding in proestrus relative to the other stages (estrus, metestrus, and diestrus combined) (Supplemental Fig. 5). There were no main effects of cycle stage or interactions with stress when proestrus was compared to the other stages (estrus, metestrus, or diestrus combined).

### Effects of CNSDS on effort-related choice behavior

**Fig. 3:**
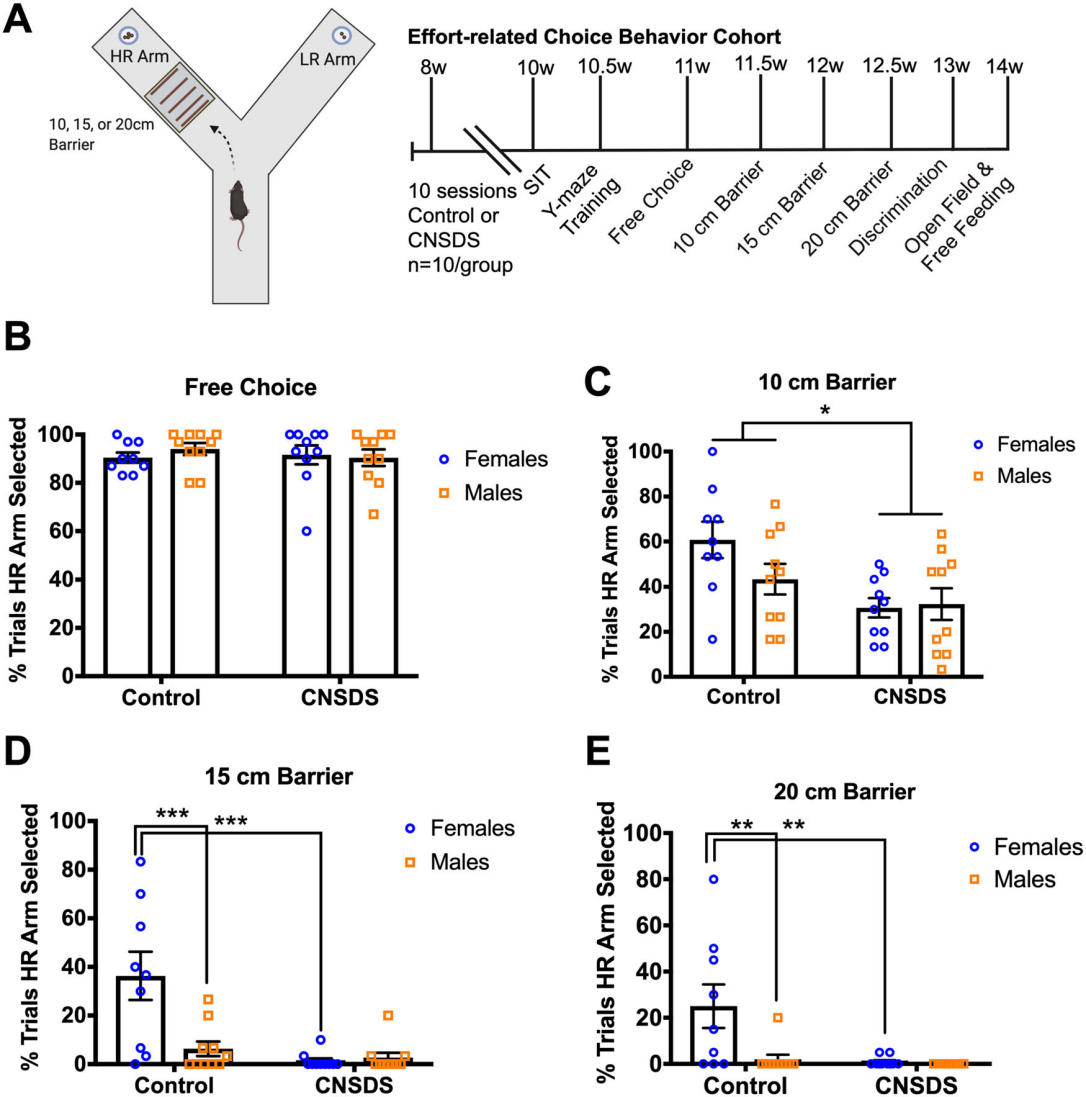
CNSDS shifts effort-related choice behavior in the Y-maze barrier task. **A** Schematic depicting the Y-maze barrier task with a barrier (10 cm, 15 cm, or 20 cm) placed in the HR arm containing 4 reward pellets versus 2 pellets in the LR arm, and timeline of CNSDS protocol and Y-maze training and barrier testing, followed by measures of free-feeding and open field behavior in Control and CNSDS male and female mice (*n* = 10/group). **B** Percent of trials selecting the HR arm in Free Choice sessions in Control and CNSDS males and females is similar. **C** Percent of trials selecting the HR arm in 10 cm Barrier sessions is reduced in CNSDS mice compared to Control. **D** Percent of trials selecting the HR arm in 15 cm Barrier sessions is reduced in CNSDS females compared to Control females, and also in Control males controlled to Control females. **E** Similarly, in the 20 cm Barrier sessions HR arm selection is reduced in CNSDS females compared to Control females and also in Control males compared to Control females. Bars are mean ± SEM. *p < 0.05; **p < 0.01; ***p < 0.001.

We next tested the effects of CNSDS on motivation in a new cohort of males and females using an effort-related choice task. In this Y-maze barrier test, mice are presented with the option of either a high effort/high reward or a low effort/low reward^28,29^. Control males (*n* = 10) and control females (*n* = 9), as well as CNSDS-exposed males (*n* = 10) and females (*n* = 10) completed the CNSDS protocol, as previously described^27^, and were trained and tested in the Y-maze barrier task (Fig. 3A). Similar to the cohort tested in the instrumental behaviors and our previous report^27^, an independent samples *t-*test (*t*(17) = 11.08, *p* < 0.0001) demonstrated that males were aggressed more frequently than females (Supplemental Fig. 6B). CNSDS did not affect body weights in males or in females (Supplemental Fig. 6A) even though randomly assigned females showed a slight difference in week 1, prior to the start of the CNSDS paradigm (*p* = 0.05).

We began with free choice trials in the Y-maze barrier task, where mice were first trained to select a high reward arm (HR) containing 4 reward pellets, versus selecting a low reward arm (LR) containing only 2 pellets. Mice were trained until they reached a 70% criterion of HR arm selection (3–5 days), after which they advanced to barrier testing sessions with 10 cm, 15 cm, or 20 cm barriers present in the HR arm. For mean percentage of HR arm selections across the 3 free choice Y-maze sessions, a two-way ANOVA with sex (male; female) and CNSDS (Control; CNSDS) as between-subjects factors revealed no main effect of CNSDS (*F*(1, 35) = 0.1526, *p* = 0.6984), no main effect of sex (*F*(1, 35) = 0.1417, *p* = 0.7088), and no interaction (*F*(1, 35) = 0.5777, *p* = 0.4523) (Fig. 3B). Thus, sex and CNSDS did not affect HR arm selection during free choice sessions, as mice selected the HR arm on most individual trials in all sessions.

A 10 cm barrier was next placed in the HR arm, and all mice completed 3 daily sessions of 2 forced trials followed by 10 free choice trials. For percentage of trials selecting the HR arm across the three 10 cm barrier test sessions, a two-way ANOVA with sex and CNSDS as between-subjects factors revealed no main effect of sex (*F*(1, 35) = 1.409, *p* = 0.2432), a main effect of CNSDS (*F*(1, 35) = 9.595, *p* = 0.0038), and no interaction *(F*(1, 35) = 2.069, *p* = 0.1592) (Fig. 3C). Thus, CNSDS reduces the number of HR arm selections and shifts choice to the LR option in both sexes when a 10 cm barrier is present in the HR arm. Importantly, body weight did not correlate with HR arm selections when a 10 cm barrier was present (Supplemental Fig. 4F). Latency to select HR and LR arms was examined by 3-way ANOVA between sex (male; female), CNSDS (Control; CNSDS), and arm (HR, LR), and revealed only a main effect of arm (*F*(1, 71) = 4.201, *p* = 0.0441) (Supplemental Fig. 2B). Thus, collapsed by sex and CNSDS, mice displayed a shorter latency when selecting the LR arm compared to when selecting the HR arm in 10 cm barrier sessions.

After completing the 10 cm barrier trials, a 15 cm barrier was placed in the HR arm, and all mice completed 3 daily sessions of 2 forced trials followed by 10 free choice trials. A two-way ANOVA with sex and CNSDS as between-subjects factors revealed a main effect of sex (*F*(1, 35) = 8.354, *p* = 0.0066), a main effect of CNSDS (*F*(1, 35) = 15.21, *p* = 0.0004), and a significant interaction (*F*(1, 35) = 9.983, *p* = 0.0033) (Fig. 3D) in the percentage of trials where the HR arm was selected. Compared to Control females, CNSDS females selected the HR arm less frequently in 15 cm barrier session (*p* < 0.0001), while Control and CNSDS males did not differ in HR arm selection (*p* = 0.8392). Also, Control females selected the HR arm more often than Control males (*p* = 0.0003), while CNSDS females and CNSDS males did not differ in HR arm selection at the 15 cm barrier height (*p* = 0.9769).

Next, a 20 cm barrier was placed in the HR arm, and all mice completed 3 daily sessions of 2 forced trials followed by 10 free choice trials. For percentage of trials selecting the HR arm across the three 10 cm barrier test sessions, a two-way ANOVA with sex and CNSDS as between-subjects factors revealed main effects of CNSDS (*F*(1, 35) = 8.402, *p* = 0.0064) and sex (*F*(1, 35) = 7.159, *p* = 0.0113), and a significant interaction (*F*(1, 35) = 6.015, *p* = 0.0193) (Fig. 3E). CNSDS females selected the HR arm less than Control females (*p* = 0.0013). Control females selected the HR arm more than Control males (*p* = 0.0021). CNSDS males and females did not differ in arm selection (*p* = 0.9841), and Control and CNSDS males did not differ in arm selection (*p* = 0.9381). In the 15 and 20 cm barrier sessions, we noticed variability in the response of control females. Interestingly, the four females with the lowest percentage of HR arm selections with the 15 cm barrier also showed the lowest percentage of HR arm selections with the 20 cm barrier (Supplemental Fig. 6C), suggesting individual variability. Taken together, these data demonstrate that CNSDS shifts arm choice in both sexes. Furthermore, as effort requirements for the high reward increased a sex difference also emerged.

Lastly, 10 cm barriers were placed in both HR and LR arms for 3 additional sessions of 2 forced choice trials followed by 10 free choice trials. For percentage of trials selecting the HR arm across the three 10 cm barrier discrimination sessions, a two-way ANOVA with sex and CNSDS as between-subjects factors revealed no main effect of CNSDS (*F*(1, 35) = 0.1295, *p* = 0.7211), no main effect of sex (*F*(1, 35) = 0.00809, *p* = 0.9288), and no interaction (*F*(1, 35) = 0.2448, *p* = 0.6238) (Supplemental Fig. 6D). This indicates CNSDS does not affect the physical ability to climb the barriers, or the ability to discriminate between high and low reward. Latency to select HR and LR arms was examined by 3-way ANOVA between sex (male; female), CNSDS (Control; CNSDS), and arm (HR, LR), and revealed no main effects of arm (*F*(1, 71) = 1.369, *p* = 0.2458), sex (*F*(1, 71) = 0.2281, *p* = 0.6344), or CNSDS (*F*(1, 71) = 1.835, *p* = 0.1798), no arm x sex interaction (*F*(1, 71) = 0.3305, *p* = 0.5607), no arm x CNSDS interaction (*F*(1, 71) = 0.1318, *p* = 0.7176), no sex x CNSDS interaction (*F*(1, 71) = 1.374, *p* = 0.245), and no 3-way interaction (*F*(1, 71) = 0.0002155, *p* = 0.9883) (Supplemental Fig. 2E). Thus, latency was not affected by sex or CNSDS, and was similar for both HR and LR arms.

Similar to the instrumental cohort, we also assessed this effort-related choice cohort in a 1-hour free feeding task and an open field test. We did not observe effects of sex or CNSDS on feeding (Supplemental Fig. 6E) or overall distance traveled in the open field (Supplemental Fig. 6F). However, as expected^27^, CNSDS did increase avoidance of the aversive center (Supplemental Fig. 6G).

## Discussion

Our understanding of whether there are sex differences in how chronic stress affects behavior has been hampered by a lack of chronic stress paradigms that are effective in both male and female mice. We recently developed a variant of social defeat, CNSDS, that we validated as effective in both sexes primarily using avoidance behaviors^27^. We also found a small effect of CNSDS in sucrose preference, a commonly used behavioral measure of anhedonia. However, sucrose preference and avoidance behaviors do not show the same level of translational validity to human assessments as outcome devaluation, progressive ratio, and effort-related choice^8,37,38^. For these behaviors, highly similar tasks with parallel data analyses and interpretations can be performed in humans and in rodents^39^. Here we found that CNSDS leads to maladaptive impairments in progressive ratio and shifts in effort-related choice selection in both male and female mice.

We had no a priori reasons to expect sex differences in any of the dependent measures we observed. Body weights were significantly correlated with VR2 lever pressing and outcome devaluation (Supplemental Fig. 4), but not with FR1 reinforcers earned, progressive ratio reinforcers earned, and percent high reward arm selections in the Y-maze 10 cm barrier sessions. We also observed main effects of sex when analyzing the VR2 and outcome devaluation data. Thus, we only analyzed males and females separately for VR2 and outcome devaluation. For all other analyses males and females were combined. During training, we saw an effect of CNSDS on FR1 lever presses but not VR2 lever presses or reinforcers earned. However, we only used a single, active lever. A more complete examination of learning and activity during training would also require analyses of inactive lever presses. CNSDS also abolished sensitivity to a devalued outcome in females but had a more nuanced attenuating effect in males. Similarly, a standard form of chronic social defeat in males reduces sensitivity for a devalued outcome^40^. However, we cannot rule out that CNSDS-exposed mice have an impaired ability to devalue the reinforcer. Therefore, in addition to reducing effort-related motivated behaviors, CNSDS also results in nuanced reward valuation deficits in both sexes.

The CNSDS effects on both progressive ratio and effort-related choice confirm a growing consensus that stress reduces motivation in effortful behaviors^8,21^^,^^41–43^. CNSDS effects in progressive ratio in both males and females are similar to previous findings showing that other chronic stressors, such as chronic corticosterone administration in males, significantly reduce motivation to lever press on progressive ratio schedules^21^^,^^43–45^. Chronic corticosterone administration to males also reduces HR arm selection as barriers increase in height in effort-related choice^41^. In addition, acute stress (1 h of restraint) in male rats reduces preference for more costly but higher rewards in an effort-discounting task^46,47^ highly similar to the effort-related choice we assessed. In this task, fixed ratio 1 lever pressing yields a 2 pellet lower reward or progressively increasing numbers of lever presses (2, 5, 10, and 20) provide a 4 pellet high reward across consecutive blocks of trials^46,47^. CNSDS, chronic corticosterone administration, and acute restraint are dramatically different stress paradigms where rodents have entirely different experiences. However, these disparate stress paradigms all result in deficits in reward processing and motivation behaviors. These completely different methodologies leading to similar results strongly indicate that stress exposure results in maladaptive reward processing and motivation.

Male and female rodents do differ in some avoidance and instrumental behaviors^48,49^, including an avoidance task requiring increased effort^50^. The effort-discounting task was also used to assess the effects of ovariectomy and estradiol replacement on motivation to expend effort for low and high rewards in female rats^36^. Ovariectomy increased, while estradiol replacement decreased higher reward selection, demonstrating a role of estradiol in influencing effort-related choice behavior in females^36^. In other tasks, male rats show a greater preference for larger rewards associated with foot shock punishment^51^ and make more risky decisions during probability discounting^52^ than female rats. Thus, male rats show a stronger bias toward larger rewards than female rats. However, we observed a nuanced sex difference in effort-related choice when effort requirements for the larger reward increased. Control females continued to select the HR arm with the 15 cm and 20 cm barriers while control males and all stressed mice shifted to the LR arm. There also was individual variability in HR arm selection within this control female group (Supplemental Fig. 6C). One interpretation of these results is that females are more likely to select higher effort/higher reward options than males, although we did not observe a comparable sex-dependent effect in progressive ratio. However, there are several alternative explanations for this sex difference. First, we cannot rule out an effect of CNSDS on behavioral flexibility. In California mice (*Peromyscus californicus*), males exposed to social defeat show impaired behavioral flexibility in a Barnes maze, while defeat does not affect female performance^53^. Thus, males may show less flexibility than females when confronted with a change in the barrier height. Second, we cannot rule out a difference in physical ability between males and females to climb barriers. However, all mice completed multiple discrimination sessions (10 cm barriers present in both HR and LR arms of the Y-maze) and responded similarly. In addition, there were no differences between groups in total distance traveled in an open field (Supplemental Fig. 6F). Third, since females are significantly smaller than males, the sex difference in the effort-related choice task could be explained as a difference in relative reinforcer value between the sexes. However, we did not see a correlation between body weight and performance in the 10 cm barrier sessions. Ultimately the sex difference we observed with the 15 cm and 20 cm barriers in the effort-related choice task will require further investigation in future studies.

We did not see any statistically significant effects of estrous cycle stage on reward behaviors or motivation to respond in either Control or CNSDS females. We also found no effects when we compared proestrus to the other stages of the estrous cycle (estrus, metestrus, and diestrus combined), even though others have described a protective effect of estrogen in a developmental stress paradigm (early postnatal maternal separation followed by adolescent social isolation)^54^. Similarly, we did not see estrous cycle stage effects on avoidance behaviors^27^. Taken together, our data support the argument that estrous cycle does not result in more variability in female behavioral traits than what is observed in male rodents^55–57^.

Outcome devaluation, progressive ratio, and effort-related choice are translationally relevant behavioral tests for studying reward processing and motivation in rodents^8,58^. Similar behaviors can be assessed in human clinical populations^59,60^. Patients diagnosed with MDD show less attribution to positive information in a reward devaluation test^61^ and are less willing to expend effort for devalued rewards^60^. In progressive ratio, patients with MDD similarly show reduced breakpoint in monetary reward tasks where greater effort is required to obtain further reward^14,62^. These findings indicate that reward processing and motivational deficits are observed in humans diagnosed with MDD or other mood disorders, and in rodents subjected to chronic stress. The Y-maze barrier task is also translationally relevant due to the analogous human EEfRT^15,16^, where MDD patients expend less effort for monetary rewards compared to healthy controls^15^. The main findings of our study are that chronic stress exposure results in maladaptive effort-related motivated behaviors. Since chronic stress can precipitate mood disorders in humans, our results examining CNSDS effects on these translationally relevant behaviors in both sexes inform our understanding of the etiology of mood disorders.

Reward- and motivation-related behavioral tasks remain understudied in preclinical research, even though they may have more translational relevance for mood disorders such as depression than approach-avoidance tasks historically related to anxiety^39^. Furthermore, mood disorders are more commonly diagnosed in women than in men, and female rodents are historically under-utilized in preclinical mood disorder research^55,63^. These results add to previous findings that established CNSDS as an effective chronic stress paradigm in both sexes using avoidance behaviors^27^ and demonstrate that chronic stress causes reward processing and motivation deficits in both sexes.

## Supplementary information

Supplemental Text

Supplemental Figure 1

Supplemental Figure 2

Supplemental Figure 3

Supplemental Figure 4

Supplemental Figure 5

Supplemental Figure 6
