# Supplementary material for: Chronic non-discriminatory social defeat stress reduces effort-related motivated behaviors in male and female mice
Source: Transl Psychiatry. 2021 Feb 15;11:125. doi: 10.1038/s41398-021-01250-9 (PMC7884699; doi:10.1038/s41398-021-01250-9)
Supplement: Supplementary file 1 — Supplemental Text [file 41398_2021_1250_MOESM1_ESM.docx]

**Supplemental Figure Legends**

**Supplemental Figure 1. Effect of stage of the estrous cycle on instrumental reward behavior in Control and CNSDS females.** (A) Estrous cycle was monitored for the females in the instrumental reward cohort throughout behavior testing. Images of proestrus, estrus, metestrus, and diestrus stages of the estrous cycle taken at 10X magnification. (B) For Fixed Ratio 1 lever presses, a two-way ANOVA with group (Control; CNSDS) and estrus stage (proestrus; estrus; metestrus; diestrus) as between-subjects factors revealed no main effect of group (*F*(1, 32) = 0.9369, *p* = 0.3403) or estrus (*F*(3, 32) = 0.01454, *p* = 0.9976), and no interaction (*F*(3, 32) = 2.69, *p* = 0.0628). (C) For lever presses in the Devalued session of Outcome Devaluation, a two-way ANOVA with group (Control; CNSDS) and estrus stage (proestrus; estrus; metestrus; diestrus) as between-subjects factors revealed no main effect of group (*F*(1, 32) = 0.01096, *p* = 0.9173) or estrus (*F*(3, 32) = 1.892, *p* = 0.1508), and no interaction (*F*(3, 32) = 0.445, *p* = 0.7225). (D) Next, for lever presses in the Valued session of Outcome Devaluation, a two-way ANOVA with group (Control; CNSDS) and estrus stage (proestrus; estrus; metestrus; diestrus) as between-subjects factors revealed a main effect of group (*F*(1, 32) = 5.92, *p* = 0.0207), no main effect of estrus (*F*(3, 32) = 1.306, *p* = 0.2895), and no interaction (*F*(3, 32) = 0.8424, *p* = 0.4808). (E) For lever presses in PROG ratio, a two-way ANOVA with group (Control; CNSDS) and estrus stage (proestrus; estrus; metestrus; diestrus) as between-subjects factors revealed a main effect of group (*F*(1, 32) = 64.27, *p* < 0.0001), no main effect of estrus (*F*(3, 32) = 1.744, *p* = 0.1778), and no interaction (*F*(3, 32) = 0.1.418, *p* = 0.2555). (F) For percent food pellet (g) eaten per body weight (g) in the free feeding behavior, a two-way ANOVA with group (Control; CNSDS) and estrus stage (proestrus; estrus; metestrus; diestrus) as between-subjects factors revealed no main effect of group (*F*(1, 32) = 0.3963, *p* = 0.5335), or estrus (*F*(3, 32) = 1.937, *p* = 0.1434), but a significant interaction (*F*(3, 32) = 3.769, *p* = 0.0201). CNSDS females in the diestrus phase of the estrous cycle consumed less compared to CNSDS females in proestrus (*p* = 0.0432) or CNSDS females in metestrus (*p* = 0.0031). Bars are mean +/- SEM. **p* < 0.05; ***p* < 0.01; ****p* < 0.001.

**Supplemental Figure 2.** (A) Latency to select HR and LR arms in Free Choice trials. (B) Latency to select HR and LR arms in 10 cm Barrier trials. (C) Latency to select HR and LR arms in 15 cm Barrier trials. (D) Latency to select HR and LR arms in 20 cm Barrier trials. (E) Latency to select HR and LR arms in Discrimination trials. Bars are mean +/- SEM. *ns: p* > 0.05 (non-significant); **p* < 0.05.

**Supplemental Figure 3.** (A) Body weights for 7 total weeks across CNSDS and instrumental reward behavior testing (n=20/group). (B) Mean CD-1 aggressions occur more frequently of males than females during CNSDS. (C) Lever presses across seven daily sessions shows CNSDS males lever press less than Control males in the final sessions, while there is no difference in females. (D) Percent body weight consumed of a food pellet of standard lab chow in a 1-hour free-feeding behavior test was higher in females than males, while CNSDS did not affect free-feeding behavior. (E) For distance travelled, a measure of overall locomotion, there were no main effects of CNSDS (*F*(1, 76) = 1.892, *p* = 0.173), or sex (*F*(1, 76) = 0.052, *p* = 0.82), and the interaction (*F*(1, 76) = 1.616, *p* = 0.2075) was non-significant. (F) For time in the center of the OFT, the main effect of stress (*F*(1, 76) = 5.24, *p* = 0.0249) was significant, while the main effect of sex (*F*(1, 76) = 0.0345, *p* = 0.853), and the interaction between factors was not significant (*F*(1, 76) = 1.991, *p* = 0.1623). Bars are mean +/- SEM. **p* < 0.05; ***p* < 0.01; ****p* < 0.001.

**Supplemental Figure 4. Correlations between body weight and behavior.** (A) Body weight was not significantly correlated to FR1 reinforcers earned (*r* = 0.1226, *p* = 0.2785). (B) Body weights were significantly correlated to (B) VR2 reinforcers earned (*r* = 0.2313, *p* = 0.0403). (C) Body weight trended but was not significantly related to lever presses in the Valued session of Outcome Devaluation (*r* = 0.2150, *p* = 0.0555). (D) Body weight was correlated with lever presses in the Devalued session of Outcome Devaluation (*r* = 0.2276, *p* = 0.0424). (E) Body weight was not correlated to PROG reinforcers earned (*r* = 0.1078, *p* = 0.3413), or (F) with percent high reward arm selections in the Y-maze 10 cm barrier sessions (*r* = 0.05465, *p* = 0.7411).

**Supplemental Figure 5. Effect of proestrus on instrumental reward behavior in Control and CNSDS females.** To examine the impact of proestrus compared to other stages of the estrous cycle on reward behaviors in the females, separate two-way ANOVAs with stress (Control; CNSDS) and estrogen (high; low) as between-subjects factors were conducted. (A) For lever presses in FR1, a two-way ANOVA revealed no main effect of CNSDS (*F*(1, 36) = 0.491, *p* = 0.488) or proestrus (*F*(1, 36) = 0.00404, *p* = 0.9497), and no interaction (*F*(1, 36) = 0.0548, *p* = 0.8162). (B) For lever presses in the Devalued session of Outcome Devaluation, a two-way ANOVA revealed no main effect of CNSDS (*F*(1, 36) = 0.001278, *p* = 0.9717) or proestrus (*F*(1, 36) = 0.0279, *p* = 0.8682), and no interaction (*F*(1, 36) = 0.734, *p* = 0.3973). (C) For lever presses in the Valued session of Outcome Devaluation, a two-way ANOVA revealed no main effect of CNSDS (*F*(1, 36) = 1.976, *p* = 0.1684) or proestrus (*F*(1, 36) = 0.0008437, *p* = 0.977), and no interaction (*F*(1, 36) = 2.37, *p* = 0.1324). (D) For lever presses in Progressive Ratio, a two-way ANOVA revealed a significant main effect of CNSDS (*F*(1, 36) = 36.44, *p* < 0.0001), no main effect of proestrus (*F*(1, 36) = 1.008, *p* = 0.322), and a non-significant interaction (*F*(1, 36) = 2.856, *p* = 0.0996). (E) For home cage feeding control, a two-way ANOVA revealed no main effect of CNSDS (*F*(1, 36) = 1.883, *p* = 0.1785) or proestrus (*F*(1, 36) = 0.03625, *p* = 0.8501), and a non-significant interaction (*F*(1, 36) = 3.677, *p* = 0.0631). Bars are mean +/- SEM. **p* < 0.05; ***p* < 0.01; ****p* < 0.001.

**Supplemental Figure 6.** (A) Body weights for 7 total weeks across CNSDS and behavior testing in the Y-maze barrier task in Control and CNSDS males and females (n=10/group). (B) Mean CD-1 aggressions are less in females than males during CNSDS. (C) Percent of trials selecting the high reward arm (HR arm) in Control females in 15 cm and 20 cm barrier sessions of the Y-maze task. (D) Percent of trials selecting the HR arm in Y-maze Discrimination sessions where 10 cm barriers were present in the HR and also LR arms are similar across groups. (E) Control and CNSDS males and females were food-deprived for 18 hours and then placed in a fresh cage with one food pellet of standard lab chow for a 1-hour free-feeding consumption test. For food consumed (g) as percentage of body weight (g), a two-way ANOVA with sex and CNSDS as between-subjects factors revealed no main effect of sex (*F*(1, 35) = 0.0159, *p* = 0.9003), no main effect of CNSDS (*F*(1, 35) = 1.312, *p* = 0.260), and no interaction (*F*(1, 35) = 0.2474, *p* = 0.622). Thus, neither sex nor CNSDS impacted free feeding behavior. (F) For locomotion in the OFT, a two-way ANOVA with sex and CNSDS as between-subjects factors revealed no main effect of CNSDS (*F*(1, 35) = 0.6059, *p* = 0.4416), no main effect of sex (*F*(1, 35) = 2.163, *p* = 0.1503), and no interaction (*F*(1, 35) = 0.3298, *p* = 0.5694). (G) For time in the center of the OFT, a two-way ANOVA with sex and CNSDS as between-subjects factors revealed a main effect of CNSDS (*F*(1, 35) – 5.905, *p* = 0.0204), no main effect of sex (*F*(1, 35) = 0.146, *p* = 0.7074, and no interaction (*F*(1, 35) = 0.0392, *p* = 0.8441). Bars are mean +/- SEM. **p* < 0.05; ***p* < 0.01; ****p* < 0.001.
