# Supplementary figures and images for: Chronic non-discriminatory social defeat stress reduces effort-related motivated behaviors in male and female mice
Source: Transl Psychiatry. 2021 Feb 15;11:125. doi: 10.1038/s41398-021-01250-9 (PMC7884699; doi:10.1038/s41398-021-01250-9)

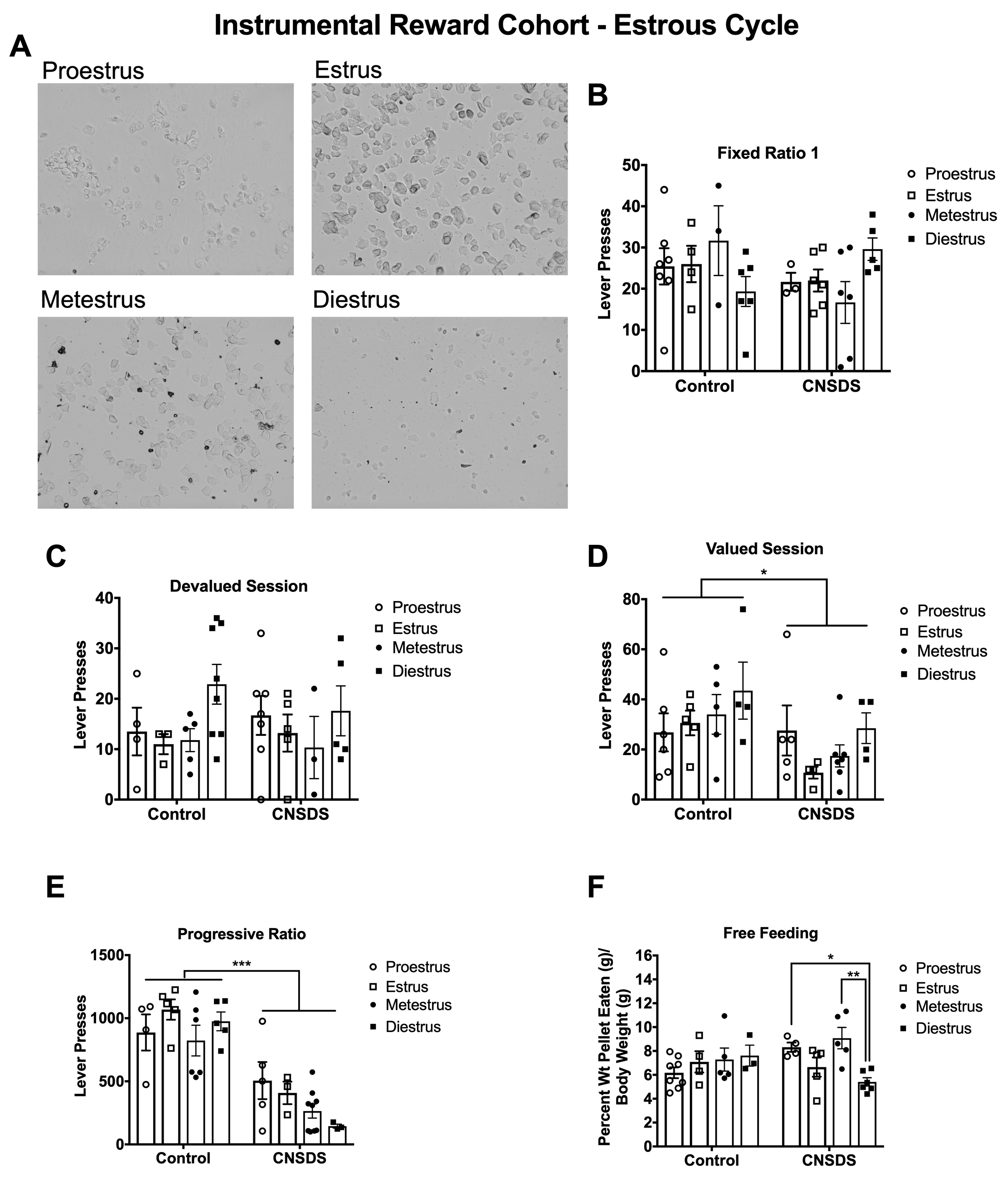

Supplement: Supplementary file 2 — Supplemental Figure 1 [file 41398_2021_1250_MOESM2_ESM.tif]

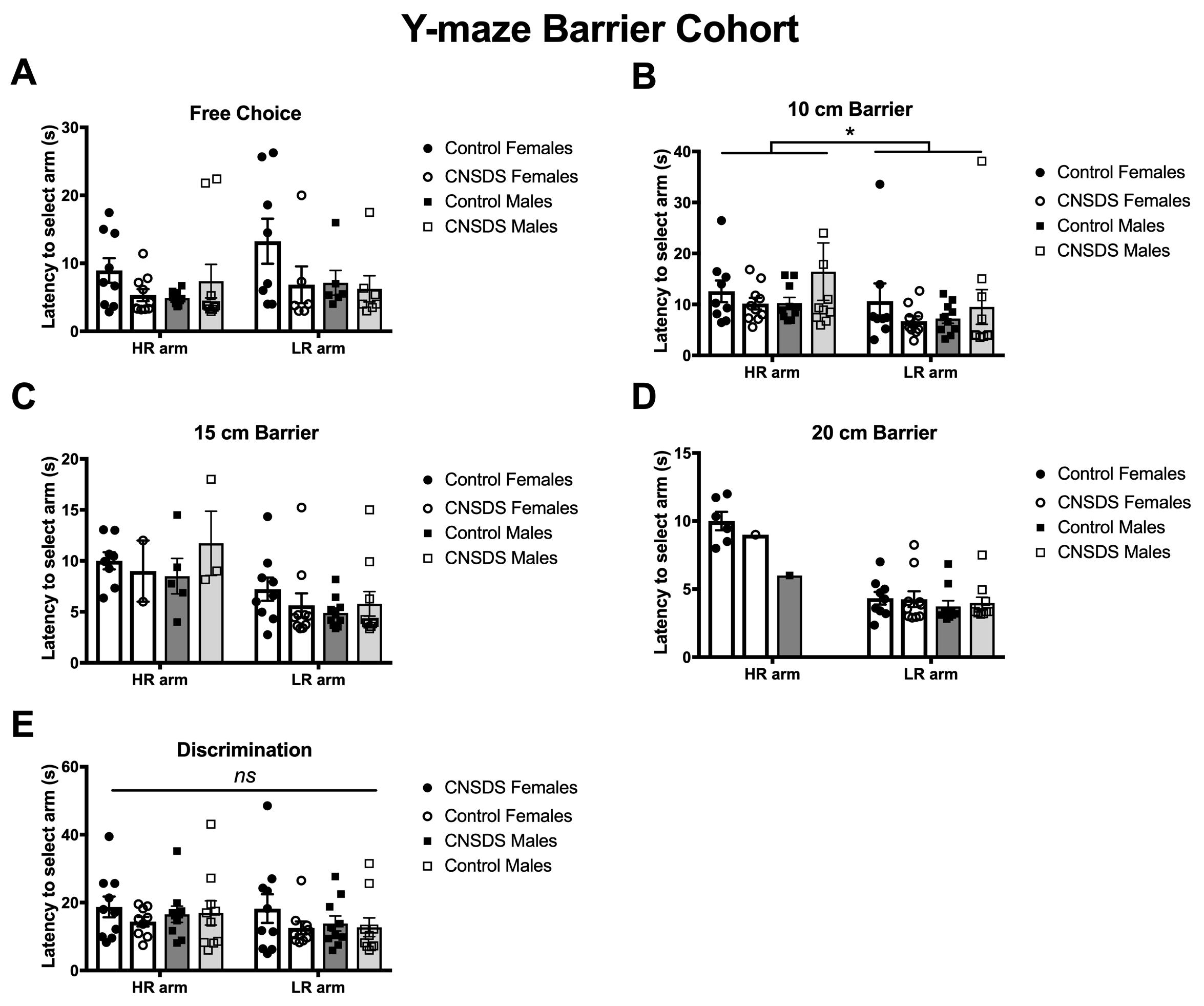

Supplement: Supplementary file 3 — Supplemental Figure 2 [file 41398_2021_1250_MOESM3_ESM.tif]

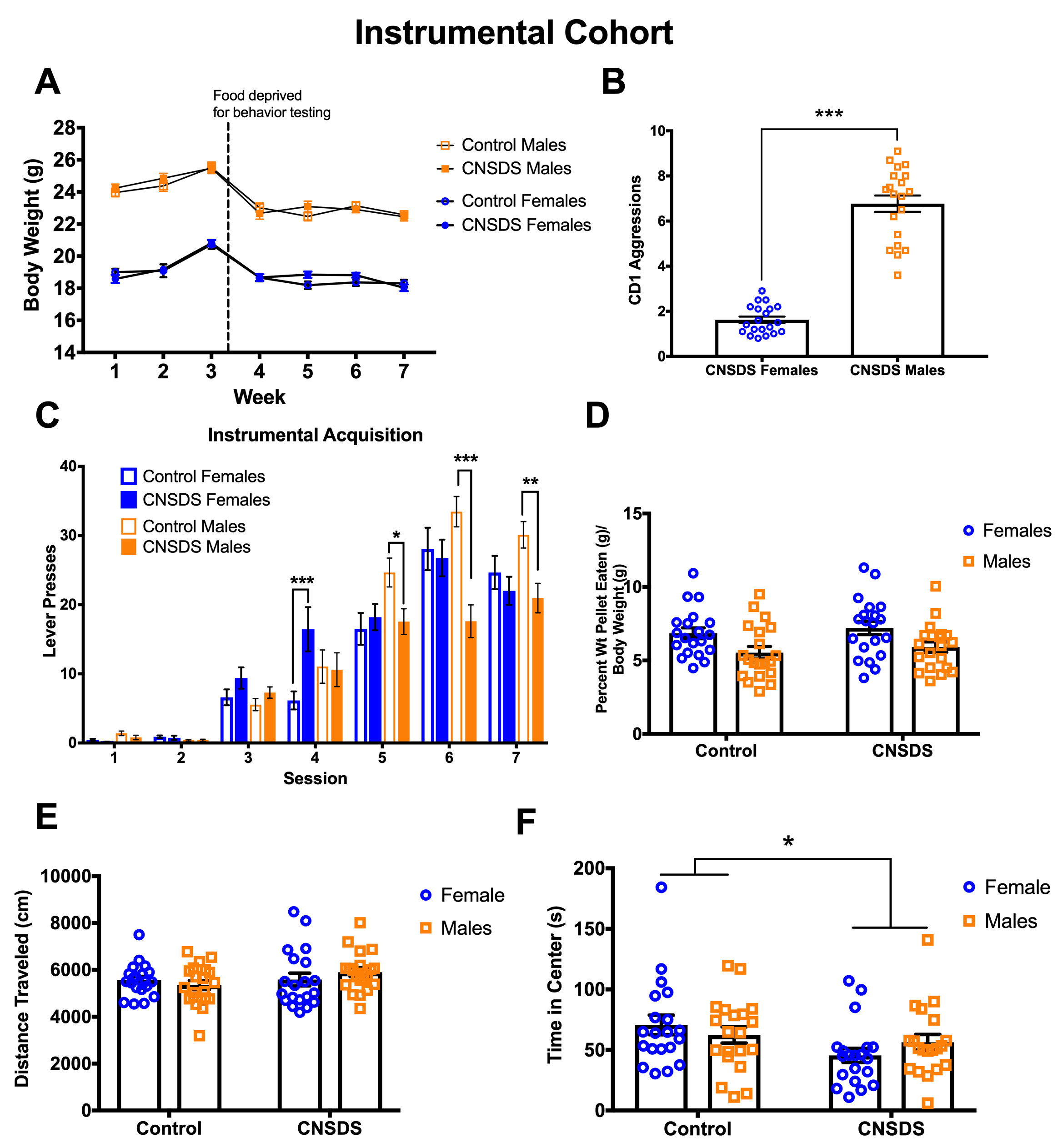

Supplement: Supplementary file 4 — Supplemental Figure 3 [file 41398_2021_1250_MOESM4_ESM.tif]

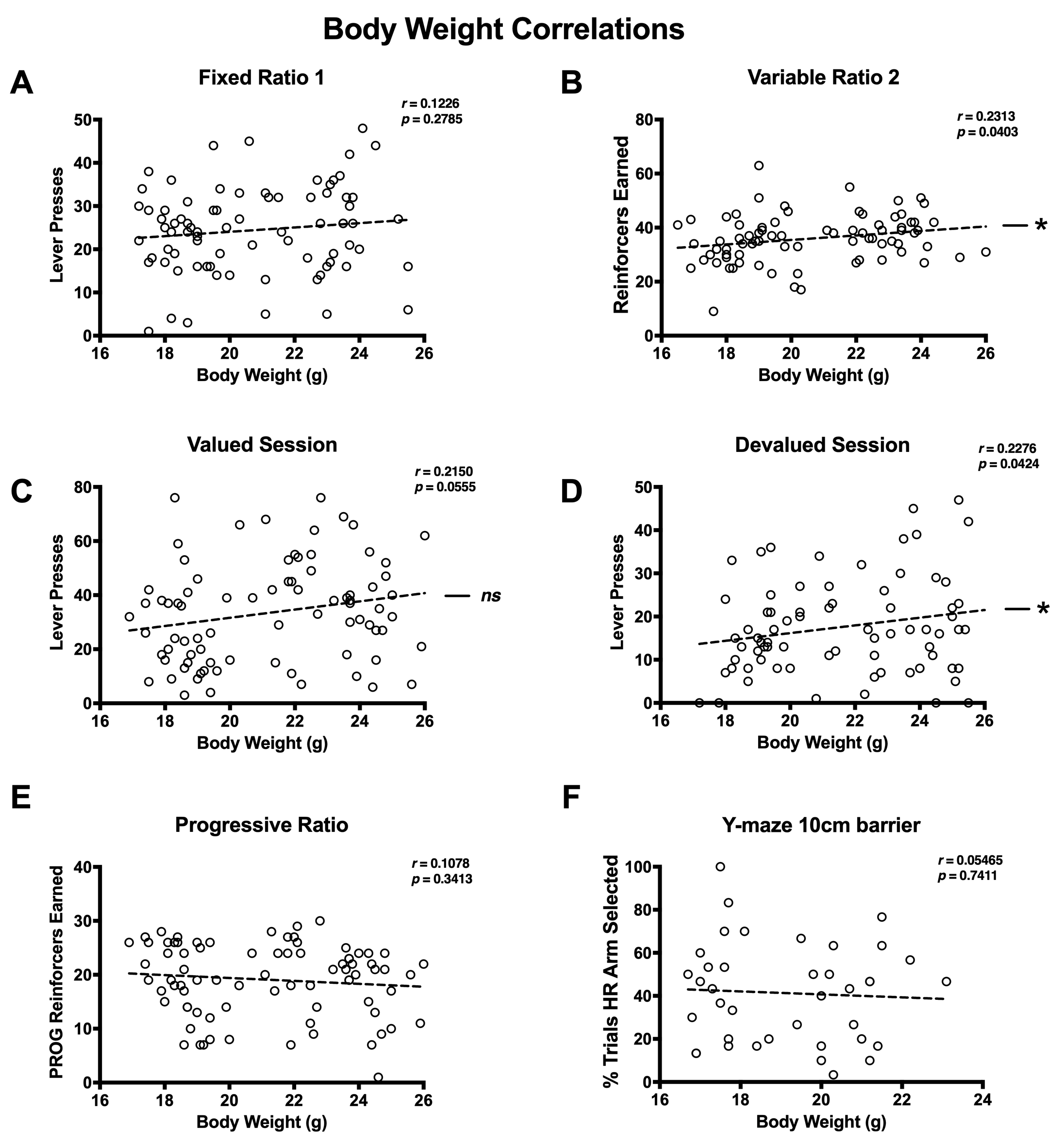

Supplement: Supplementary file 5 — Supplemental Figure 4 [file 41398_2021_1250_MOESM5_ESM.tif]

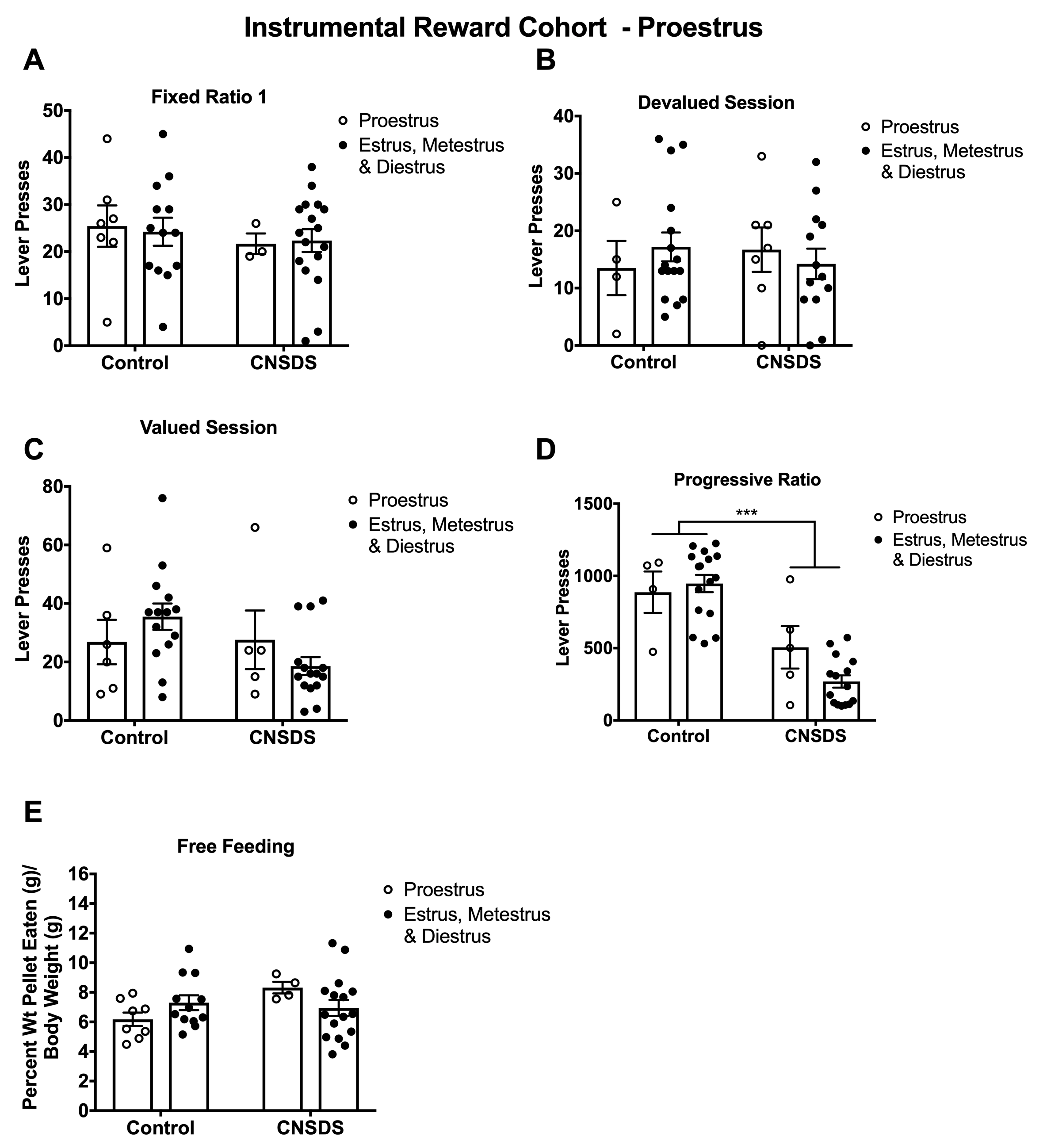

Supplement: Supplementary file 6 — Supplemental Figure 5 [file 41398_2021_1250_MOESM6_ESM.tif]

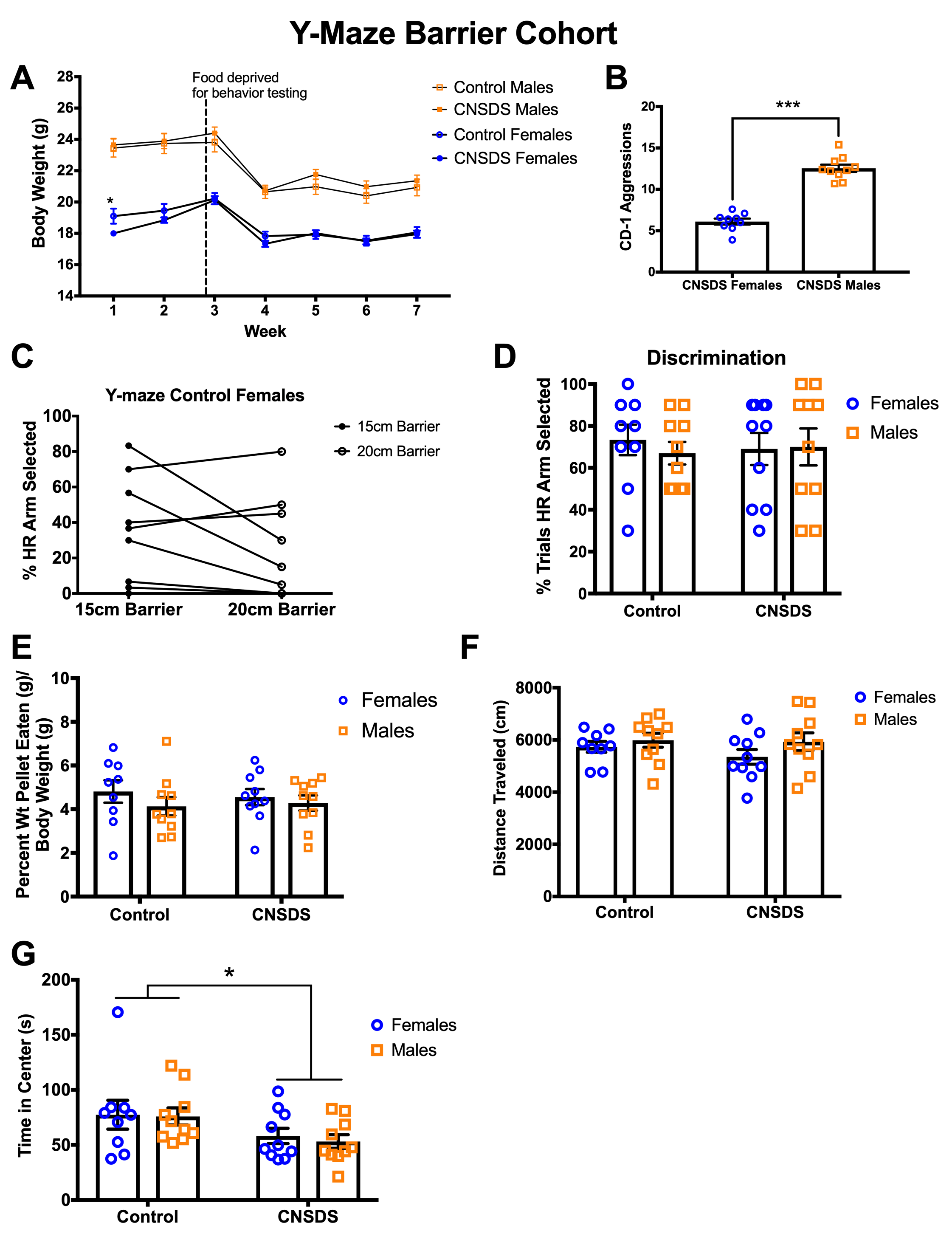

Supplement: Supplementary file 7 — Supplemental Figure 6 [file 41398_2021_1250_MOESM7_ESM.tif]
